# Importance of scientific collaboration in contemporary drug discovery and development: a detailed network analysis

**DOI:** 10.1186/s12915-020-00868-3

**Published:** 2020-10-13

**Authors:** Feixiong Cheng, Yifang Ma, Brian Uzzi, Joseph Loscalzo

**Affiliations:** 1 Genomic Medicine Institute, Lerner Research Institute, Cleveland Clinic, Cleveland, OH 44195 USA; 2 Department of Molecular Medicine, Cleveland Clinic Lerner College of Medicine, Case Western Reserve University, Cleveland, OH 44195 USA; 3 Case Comprehensive Cancer Center, Case Western Reserve University School of Medicine, Cleveland, OH 44106 USA; 4 Department of Statistics and Data Science, Southern University of Science and Technology, Shenzhen, 518055 China; 5 Northwestern Institute on Complex Systems (NICO) and Kellogg School of Management, Northwestern University, Evanston, IL 60208 USA; 6 Department of Medicine, Brigham and Women’s Hospital, Harvard Medical School, 75 Francis St., Boston, MA 02115 USA

**Keywords:** Cardiovascular disease, Collaboration network, Drug discovery, Network analysis, PCSK9, Scientific collaboration, TNF inhibitors

## Abstract

**Background:**

Growing evidence shows that scientific collaboration plays a crucial role in transformative innovation in the life sciences. For example, contemporary drug discovery and development reflects the work of teams of individuals from academic centers, the pharmaceutical industry, the regulatory science community, health care providers, and patients. However, public understanding of how collaborations between academia and industry catalyze novel target identification and first-in-class drug discovery is limited.

**Results:**

We perform a comprehensive network analysis on a large scientific corpus of collaboration and citations (97,688 papers with 1,862,500 citations from 170 million scientific records) to quantify the success trajectory of innovative drug development. By focusing on four types of cardiovascular drugs, we demonstrate how knowledge flows between institutions to highlight the underlying contributions of many different institutions in the development of a new drug. We highlight how such network analysis could help to increase industrial and governmental support, and improve the efficiency or accelerate decision-making in drug discovery and development.

**Conclusion:**

We demonstrate that network analysis of large public databases can identify and quantify investigator and institutional relationships in drug discovery and development. If broadly applied, this type of network analysis may help to enhance public understanding of and support for biomedical research, and could identify factors that facilitate decision-making in first-in-class drug discovery among academia, the pharmaceutical industry, and healthcare systems.

## Background

A recent study estimates that in 2015, it costs the pharmaceutical industry $2.6 billion to develop a new US Food and Drug Administration (FDA)-approved drug [1]. This high cost is, in part, a consequence of the collaborative complexity of the drug development process [2]. Contemporary drug discovery and development reflect the work of teams of individuals from academic centers, the pharmaceutical industry, the regulatory science community, health care providers, and patients. Within academia, current drug discovery and development involve scientific collaborations between laboratories, is multidisciplinary, and almost invariably crosses inter-institutional boundaries among industrial and academic institutions.

Scientific collaboration is more strikingly prevalent today than it was several decades ago [3, 4]. In many important areas of biomedical research, the scientific process increasingly involves catalyzing collaborative efforts that bring together investigators with diverse scientific backgrounds and perspectives to solve complex biomedical problems that benefit from an interdisciplinary or multidisciplinary approach [5, 6]. Public understanding of how collaborations between academia and industry result in novel target identification and first-in-class drug discovery is limited [7]. Furthermore, whether team-driven human genetic studies, for example, accelerate target identification and which type of collaborative arrangement will maximize the efficiency of drug discovery, remain unclear [8–10]. Here, we analyze a large scientific corpus of collaboration and citation networks to quantify the success trajectory of drug development using proprotein convertase subtilisin/kexin type 9 (PCSK9) and its inhibitors as a case study, as well as phosphodiesterase type 5 (PDE5) inhibitors, hydroxymethylglutaryl (HMG)-CoA reductase inhibitors, and tumor necrosis factor (TNF) inhibitors as additional examples of different, commonly used drug classes.

## Results

### Publications record the trajectory from the discovery of PCSK9

**Fig. 1:**
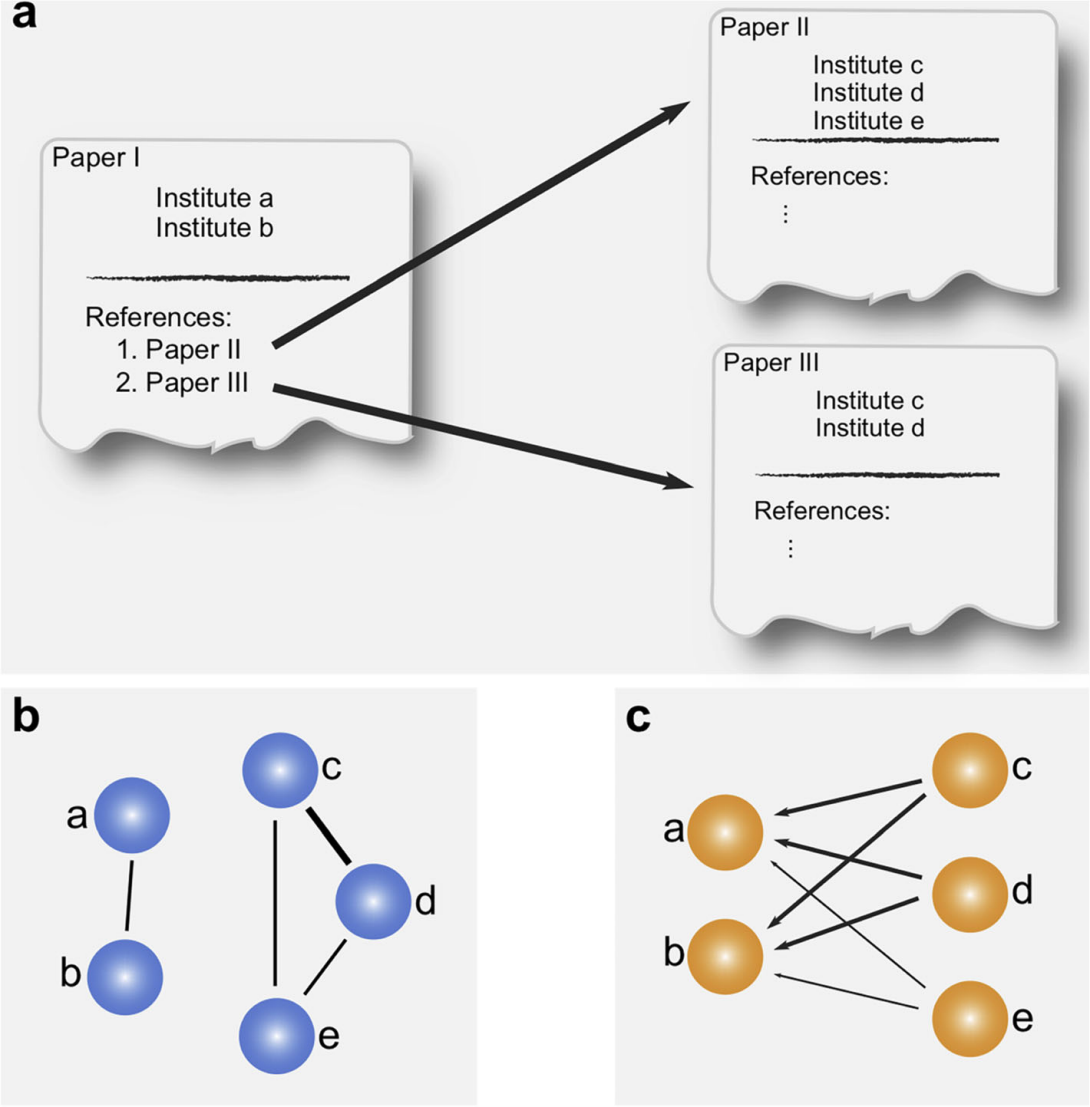
Projecting paper institutions and references to the institutional collaboration network and the institutional knowledge flow network. **a** Paper I written by authors from institution *a* and *b* cite paper II written by authors from institution *c*, *d* and *e*, and paper III written by authors from institution *c* and *d*. **b** Collaborations among the five institutions based on the affiliations in the three papers. Link strength between institution *c* and *d* is 2; other link strengths are 1. **c** Directed links indicate the knowledge flows from institution *c*, *d*, and *e* to institution *a* and *b*; links from *c/d* to *a/b* have weight 2 and links from *e* to *a/b* have weight 1

**Fig. 2:**
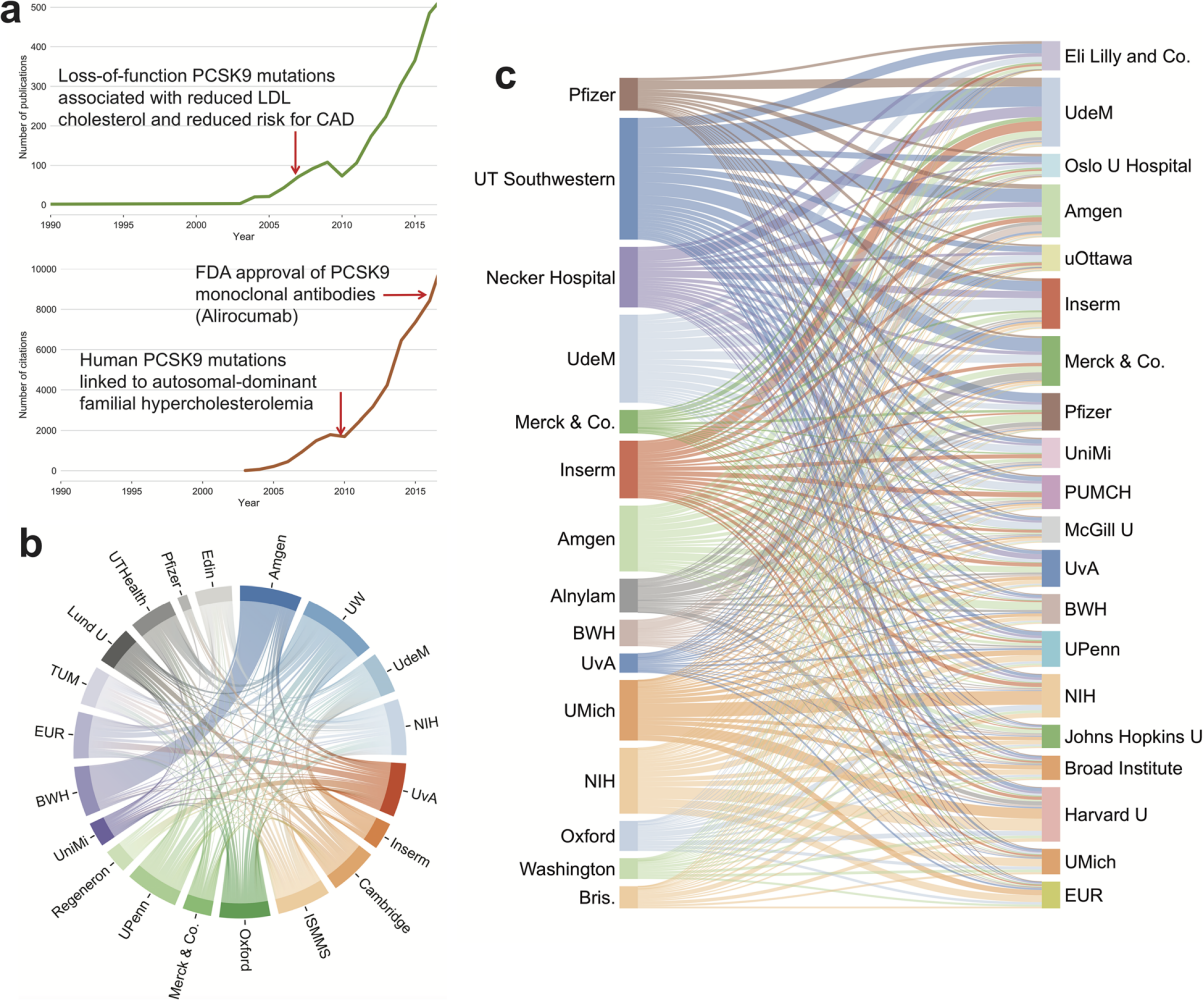
PCSK9 target discovery and development network analysis. **a** The number of publications and number of citations for PCSK9 papers by year. **b** Collaboration network in the discovery of PCSK9 for the top 20 institutions. Stripe width between institutions corresponds to the collaboration strength, i.e., the number of cases in which the two institutions collaborate. **c** The citation flow from cited papers (left) to citing papers (right). Stripe width from institutions on the left to institutions on the right corresponds to the number of cases in which papers from institutions on the left are cited by papers from institutions on the right

We utilized a comprehensive analysis that integrates large-scale publicly accessible scientific datasets (Fig. 1 and cf. the “Methods” section). We used the Microsoft Academic Graph (MAG) database [11], which contains 170,099,684 publications dating from 1900 to 2017. In MAG, papers’ topics are classified using artificial intelligence and semantic understanding of content [12]. Each scientist’s institution(s) is (are) identified using the affiliation information within the publication, with the specific commercial and academic institutions manually identified (Fig. 1). In the primary case study, we assembled all papers related to PCSK9 [13], with the tag “PCSK9” and its aliases (Methods), and identified 2,675 publications and 50,513 additional relevant citations from 1900 to 2017. From these papers, we successfully presented the full trajectory of PCSK9’s discovery and development (Fig. 2a). Importantly, we found the same trajectory after excluding self-citations (Additional file 1: Fig. S1). For example, a human genetic study in 2003 first reported that gain-of-function PCSK9 mutations led to hypercholesterolemia [14], after which the number of papers and citations began to increase. Three years later, a second human genetic study reported that PCSK9 loss-of-function mutations reduced low-density lipoprotein cholesterol (LDL-c) and protected against coronary heart disease [15].

### Collaboration network structure from the discovery of PCSK9 and its inhibitors

We next inspected the collaboration network between institutions; in the network, we regarded each institution as a node, with the weighted links between institutions reflecting the number of papers on which collaboration occurred (Fig. 1). By referring to the institutions in all PCSK9 papers, we found that the development of the PCSK9 field involved the collaborations of 9,286 scientists distributed among 4,203 institutions worldwide over the last two decades. For example, Amgen investigators published 548 PCSK9-related papers in the last two decades, followed by the University of Montreal (UdeM) with 452 papers and Inserm with 414 papers. Forty percent of the collaborations involved intra-institutional co-investigators (i.e., scientists within the same institutions), while the remaining 60% of collaborations involved inter-institutional co-investigators (i.e., scientists in different institutions). Among the inter-institutional collaborations, 20% involved pharmaceutical companies, highlighting the critical, but non-exclusive, role of the industry in drug target discovery.

In Fig. 2b, we show the relationships among the top 20 most collaborative institutions (according to their degree in the collaboration network). We note that Amgen and Brigham and Women’s Hospital/Harvard Medical School have a strong collaborative tie, as do other strongly collaborative institutions such as the University of Montreal (UdeM) and Inserm and the University of Amsterdam (UvA) and Regeneron. The collaboration between institutions is not uniform, with 6% of the top institutions accounting for 90% of the collaboration weights in the network, illustrating that a small number of institutions dominate the research.

**Fig. 3:**
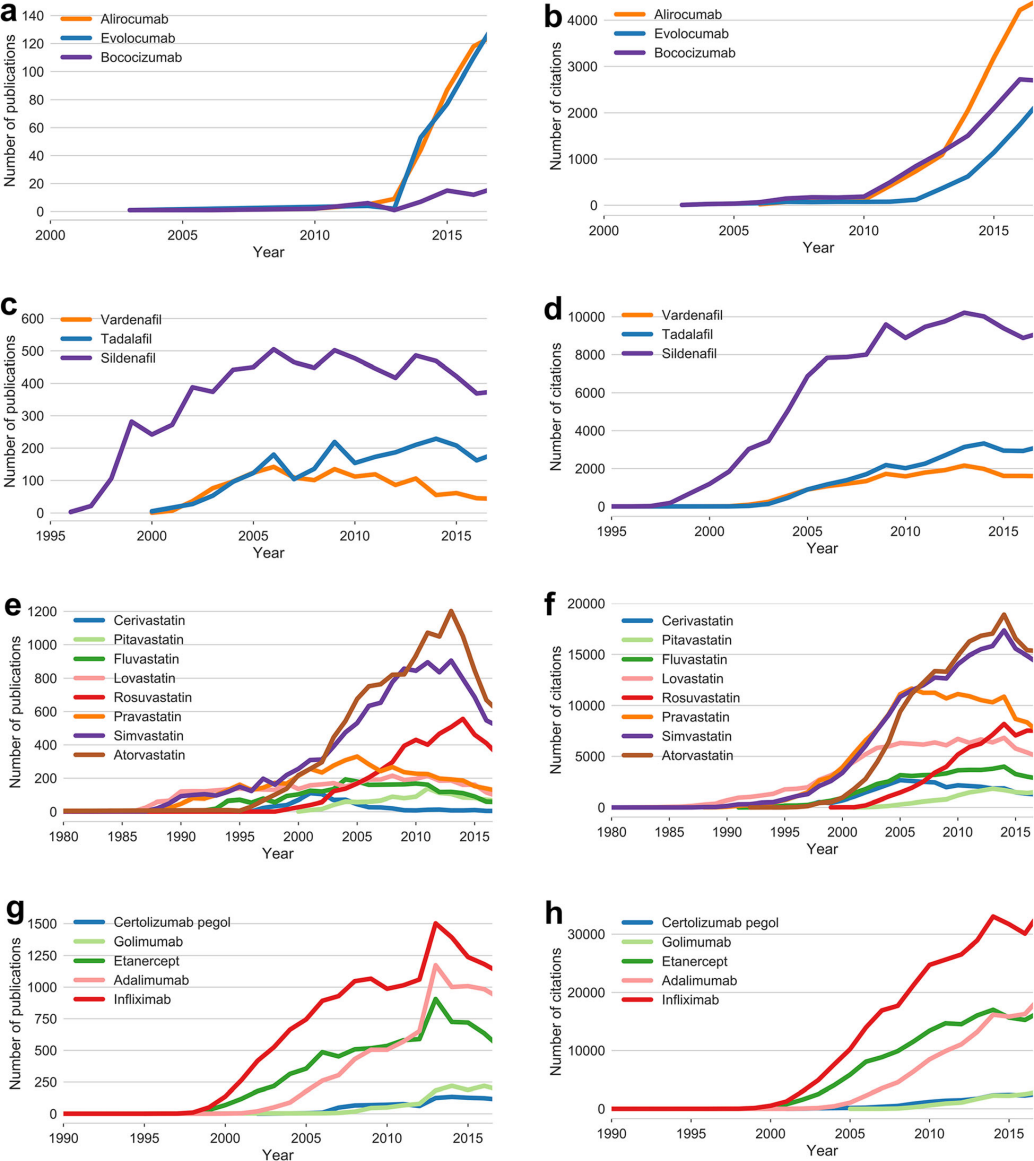
Publication and citation growth. The number of annual publications (column **a**, **c**, **e**, **g**) and the number of annual citations (column **b**, **d**, **f**, **h**) for **a**, **b** 3 PCSK9 inhibitors (alirocumab, evolocumab, and bococizumab), **c**, **d** 3 PDE5 inhibitors (vardenafil, tadalafil, and sildenafil), **e**, **f** 8 HMG-CoA reductase inhibitors (cerivastatin, pitavastatin, fluvastatin, lovastatin, rosuvastatin, pravastatin, simvastatin, and atorvastatin,), and **g**, **h** 5 TNF inhibitors (certolizumab pegol, golimumab, etanercept, adalimumab, and Infliximab). In total, 170,099,684 publications dating from 1900 to 2017 were analyzed (see the “Methods” section)

**Fig. 4:**
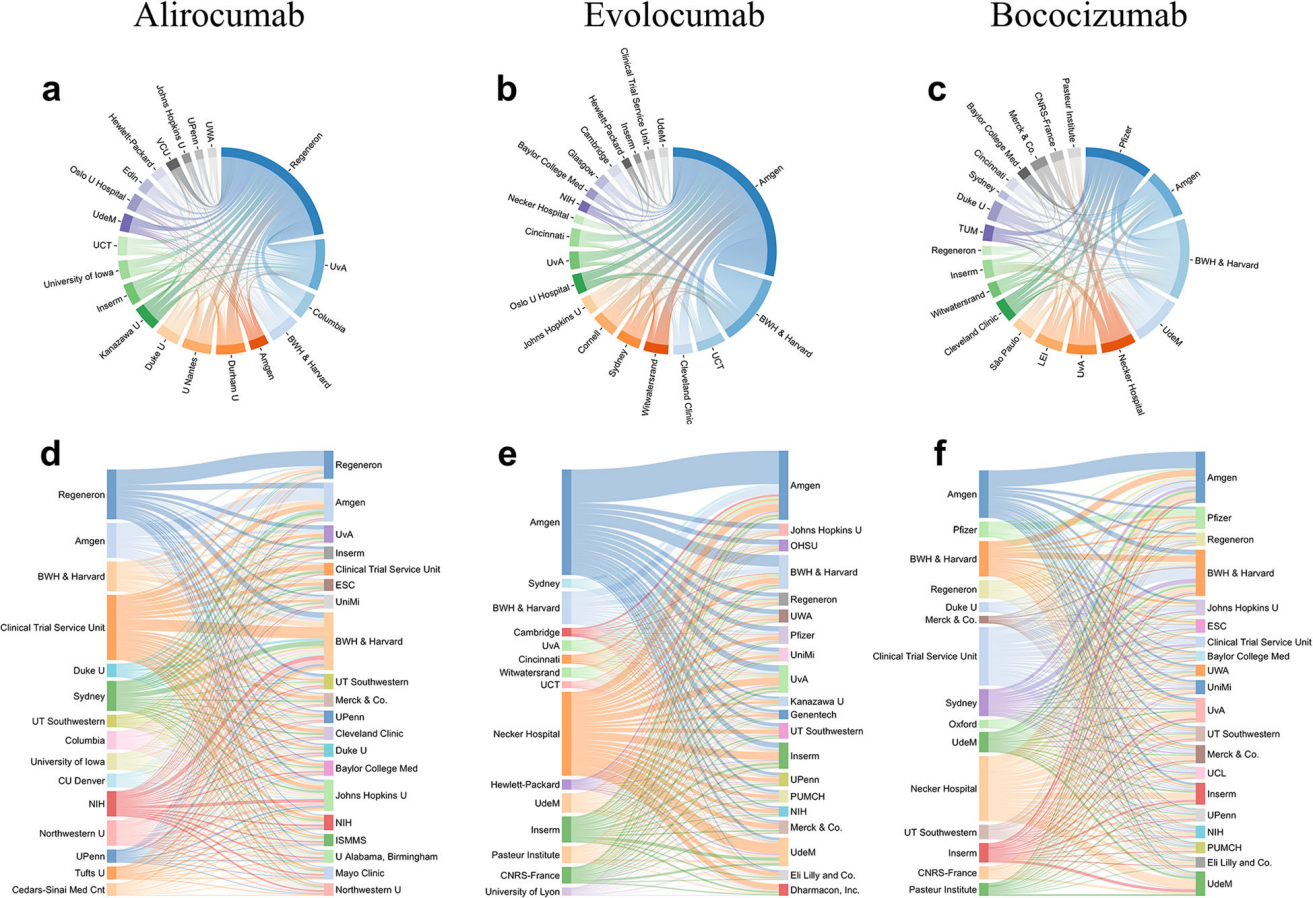
PCSK9 inhibitors network analysis. **a**–**c** Collaboration network for the top 20 institutions. Stripe width between institutions corresponds to the collaboration strength. **d**–**f** The citation flow for the top institutions. Stripe width from institutions on the left to institutions on the right corresponds to the number of cases in which papers from institutions on the left were cited by papers from institutions on the right

For comparison, we further investigated the collaboration networks for three specific PCSK9 inhibitors (Fig. 3): two recently FDA-approved drugs (alirocumab and evolocumab) and one failed drug (bococizumab). Alirocumab (trade name Praluent, Sanofi Aventis), a PCSK9 inhibitor monoclonal antibody, was approved by the FDA on July 24, 2015, for the treatment of patients with heterozygous familial hypercholesterolemia or atherosclerotic cardiovascular disease based on five double-blind placebo-controlled trials that enrolled 3,499 patients. The studies related to alirocumab involved 1,407 different investigators who published 403 papers and listed 908 different institutional affiliations (Figs. 3 and 4a). Evolocumab (trade name Repatha, Amgen), the second human monoclonal antibody, was approved by the FDA on August 27, 2015, as an adjunct treatment to diet and maximally tolerated statin therapy in adults with heterozygous or homozygous familial hypercholesterolemia, or those with clinical atherosclerotic cardiovascular disease [16]. On December 1, 2017, the FDA approved evolocumab to prevent myocardial infarction, stroke, and coronary revascularization in adults with established cardiovascular disease based on the 27,564-patient FOURIER cardiovascular outcome study [17]. Specifically, evolocumab reduced the risk of myocardial infarction by 27%, the risk of stroke by 21%, and the risk of coronary revascularization by 22% [17]. The collaboration network leading to the development of evolocumab included 1,185 different investigators who published 400 papers and listed 680 different institutional affiliations (Fig. 4b). Bococizumab, a PSCK9 monoclonal antibody developed by Pfizer, was withdrawn in November, 2016, owing to a lack of significant clinical benefit to patients (NCT01975389). The collaboration network leading to bococizumab included only 346 investigators across 173 different institutions who published 66 papers (Fig. 4c). Comparing the three inhibitors’ collaboration networks to that for PCSK9 as a disease target (Fig. 2b), we found that the collaboration networks for PCSK9 inhibitor development are dominated by pharma, with Regeneron, Amgen, and Pfizer contributing much more in the development of the three inhibitors than academic institutions (Fig. 4). Furthermore, for a comprehensive comparison, we calculated the network indices for each of the collaboration networks (Table 1). We validated that the collaboration networks for the three PCSK9 inhibitors have higher industrial participation (> 40%) than the collaboration networks for the PCSK9 as a disease target (20%). Compared to alirocumab and evolocumab, bococizumab (a failed drug) has a larger average clustering and a larger value of “[f]raction of top institutions accounting for 90% collaborations” (Table 1), suggesting that the more narrowly defined collaborative groups involved in this follow-on drug and also within the collaboration network are less likely to support successful collaboration in drug development.

**Table 1:** Characteristics of collaboration networks for four classes of drugs

Drug name	Target class	No. of papers	No. of authors	No. of institutions	Fraction with inter-institutional collaboration	Fraction with industrial participation	Average clustering	Assortativity	Fraction of top institutions with 90% or greater collaborations
Alirocumab	PCSK9 inhibitors	403	1407	908	0.72	0.429	0.015	− 0.087	0.126
Bococizumab		66	346	173	0.73	0.465	0.047	− 0.057	0.347
Evolocumab		400	1185	680	0.63	0.509	0.006	− 0.075	0.153
Sildenafil	PDE5 inhibitors	8018	25,171	12,659	0.39	**0.128**	**0.009**	− 0.018	**0.043**
Tadalafil		2468	7918	4556	0.45	0.236	0.012	− 0.055	0.073
Vardenafil		1464	4407	2556	0.41	0.240	0.012	− 0.022	0.098
Atorvastatin	Statins (HMG-CoA reductase inhibitors)	13,478	42,607	22,201	0.49	0.162	0.003	0.060	0.024
Cerivastatin		722	2725	1416	0.38	0.150	**0.014**	0.066	**0.129**
Fluvastatin		2848	9722	5112	0.36	0.131	**0.020**	0.021	0.079
Lovastatin		4554	15,168	7679	0.39	0.139	0.008	0.023	0.068
Pitavastatin		1228	4660	2212	0.37	**0.052**	0.007	0.047	0.076
Pravastatin		5356	18,214	8403	0.45	0.108	0.002	**−0.016**	0.047
Rosuvastatin		5285	17,718	9242	0.59	0.134	0.003	0.095	0.037
Simvastatin		12,738	43,187	21,691	0.85	**0.067**	0.001	0.101	0.007
Adalimumab	TNF inhibitors	8756	30,178	19,734	0.61	0.126	0.003	0.026	0.020
Certolizumab pegol		1052	3639	2085	0.89	**0.024**	0.004	**0.067**	0.052
Etanercept		8521	28,705	15,002	0.55	0.187	0.006	− 0.024	0.030
Golimumab		1285	4810	2980	0.69	**0.430**	0.006	− 0.032	**0.073**
Infliximab		16,371	52,436	31,727	0.55	0.134	0.002	− 0.012	0.015

Note: The significant changes of network characteristics are highlighted by bold text. The detailed descriptions for network indices are provided in the “Methods” section

### Heterogeneous collaboration patterns in drug discovery

Recent studies have suggested that network-based analysis of citations from literature data offers potential evidence for the novelty, disruption, and success of scientific research [4, 18], including drug discovery [7]. We posited that this complex collaboration network may help us identify factors that improve the efficiency or accelerate decision-making in drug discovery and development. To answer this question, we created and compared additional collaboration network structures for three additional types of drugs classes: (i) three phosphodiesterase type 5 (PDE5) inhibitors (Additional file 1: Fig. S2), (ii) eight statins HMG-CoA reductase inhibitors, (Additional file 1: Fig. S3), and (ii) five TNF inhibitors (Additional file 1: Fig. S4), and calculated their network indices (Table 1). We found several interesting patterns across different network indices. For example, among the three PDE5 inhibitors (vardenafil, tadalafil, and sildenafil), sildenafil showed lower industrial participation than the other two, a lower clustering coefficient (0.009), and collaborator research that was dominated by leading institutions (4% of the leading institutions account for 90% of the collaboration weights, Table 1). Among the 8 HMG-CoA reductase inhibitors, pitavastatin and simvastatin have lower industrial participation and cerivastatin and fluvastatin have higher than average clustering, while pravastatin has a negative assortativity (Table 1). This negative assortativity means that institutions tend to collaborate with other institutions with very different degrees within the network. Lovastatin (trade name Mevacor), the first statin approved by the US FDA, showed a moderate number of publications compared with other newer statins, suggesting low literature bias. Atorvastatin (trade name Lipitor), a first-line statin for the prevention of cardiovascular diseases, showed the highest number of publications and the highest industrial participation. Although atorvastatin is a highly successful drug developed by Pfizer, Pfizer is not the top-most institution involved in atorvastatin’s collaboration network (Additional file 1: Figs. S3a and S3e), suggesting high heterogeneity of collaboration relationships among institutions in successful drug development. Among the 5 TNF inhibitors, certolizumab pegol had lower than average industrial participation, while golimumab had greater than average industrial participation (Additional file 1: Fig. S4). From the comparisons, we can see that although the network indices vary across drug classes, they successfully capture potential network features involved in collaborative drug discovery among academic and industrial institutions for specific drug classes. Equally important, among all of the network indices, no clear and consistent patterns emerged that crossed drug classes. For example, first-in-class drugs did not exclusively derive from industry or academia, nor did follow-on drugs invariably derive from purely intra-institutional industry collaborations. We interpret these findings to indicate that collaboration networks underlying drug development evolve from local investigator or institutional interests that are driven by intellectual and cultural champions.

### The contribution from knowledge flow networks

We further learned that the knowledge contribution of successful drug target identification is complex, hierarchical, and interdisciplinary. Specifically, we built an institutional citation network by tracing the citations between institutions based on the affiliation information of authors and the cited references. Citations between research institutions show the patterns of knowledge flow in developing PCSK9 as a drug target (Fig. 2c). Importantly, knowledge does not spread randomly from one institution to another. In Fig. 2c, we show the knowledge flux between pairs of institutions, with knowledge disseminating from the cited institutions (left) to the citing institutions (right). In total, 4.6% of the citation flow is within the same institutions, i.e., papers cite other papers derived from investigators in the same institution. Specifically, for example, the University of Montreal (UdeM) shows citations spreading to more than 1,000 institutions worldwide (only the top 20 are shown in Fig. 2c); however, 11.9% of the citations come from the institution itself, as was the case for Amgen, with a value of 10.1%. Other institutions show different knowledge flow patterns. For example, NIH and Brigham and Women’s Hospital/Harvard Medical School spread knowledge relatively evenly to certain leading institutions, with only 4.1% and 4.0% of the knowledge disseminated internally, respectively. We also show the knowledge flow patterns for PDE5 inhibitors, HMG-CoA reductase inhibitors, and TNF inhibitors for comparison and validation of these findings (Additional file 1: Figs. S2-S4).

## Discussion

Here, we demonstrate that network analysis of large public databases can identify and quantify investigator and institutional relationships in drug discovery and development. We also show different collaboration patterns in drug discovery based on publication history for four classes of commonly used drugs. By comparing the three PCSK9 inhibitors, we found that the collaboration network with many narrow collaboration groups, or groups that are less concentrated in the top 90%, may be a potential proxy for failure. We demonstrate how knowledge flows between institutions to highlight the underlying (and often unnoticed) contributions of many different institutions in the development of a new drug. While this analysis is not comprehensive, it does show that none of these highly successful drug classes identified and developed drug candidates as a purely internal process within a single institution. Collaboration is not only commonplace, but also likely essential for success, requiring academia-industry interaction and cooperation. Recent studies suggest that citation and collaboration networks from literature data provide evidence for impact, novelty, and success for academic-industry partnership and innovation relating to the biomedical/pharmaceutical industry [19–21].

## Conclusions

Scientific collaboration is more strikingly prevalent today than it was several decades ago. For example, contemporary drug discovery and development reflects the work of teams of individuals from academic centers, the pharmaceutical industry, the regulatory science community, health care providers, and patients. However, public understanding of how collaborations between academia and industry result in novel target identification and first-in-class drug discovery is limited. In this study, we performed a comprehensive network analysis on a large scientific corpus of collaboration and citations. We demonstrate that network analysis of large public databases can identify and quantify investigator and institutional relationships in drug discovery and development. If broadly applied, this type of analysis may help to enhance public understanding of and support for biomedical research, and may identify factors that facilitate decision-making in drug discovery among academia, the pharmaceutical industry, and healthcare systems.

## Methods

### Data resources

We used the MAG database [11], which contains 170,099,684 publications from 1900 to 2017. In total, we extracted and analyzed 97,688 papers, as well as their 1,862,500 citations, and all of the affiliation information in each paper from MAG. We used the machine learning-based tags to identify the papers that study a specific drug annotated by generic name and Medical Subject Headings (MeSH) vocabularies [22]. We combined the aliases from the human gene database, GeneCards (https://www.genecards.org/cgi-bin/carddisp.pl?gene=PCSK9), and checked them manually. For PCSK9, we considered its aliases, such as proprotein convertase subtilisin/kexin type 9, FH3, HCHOLA3, LDLCQ1, NARC-1, NARC1, PC9, FHCL3, and searched each of the tags in MAG. The papers’ affiliation(s) are identified using all the authors’ affiliations within the paper, and affiliations are also well identified and linked to the official links and Wikipedia links in the database, for example, the Amgen: https://academic.microsoft.com/institution/1320553840. We manually identified the industrial institutions and the academic institutions.

### Construct collaboration and knowledge flow network

We constructed institution-level collaboration and citation networks. In the collaboration network, each node is an institution, and links with weights indicate the collaboration strength between the two institutions, i.e., the number of cases with both of the institutions appearing within the same paper. The citation flow network is a directed network, each node is an institution, and a link (edge) from institution *a* to institution *b* weighs the cases when paper affiliated *b* cites paper affiliated *a*. See Fig. 1 for the illustrative example.

### Definitions of network indices

We investigated four commonly used network indices to quantify the structure of collaboration networks. All of the following indices are defined on the whole weighted collaboration network.

#### Fraction of industrial participation

In the collaboration network, we selected all the weighted links whose connected nodes contain corporate entities and calculated the fraction of link weights associated with corporate entities over the sum of the all link weights, i.e.,

1 $$ \raisebox{1ex}{$\sum \limits_{i\ \mathrm{or}\ j\in \mathrm{Corporates}}{w}_{ij}$}\!\left/ \!\raisebox{-1ex}{${\sum}_{i,j}{w}_{ij}$}\right. $$

where *w*_*ij*_ is the collaboration strength between institution *i* and institution *j*.

#### Average clustering

This parameter is a measurement of the degree to which nodes in a graph tend to cluster together. A larger average clustering means the nodes tend to form triplets in the network. Clustering is often defined with respect to a node, and the average clustering of a network is the average over all nodes in the network. For weighted network, there are several ways to define clustering; here, we used the one defined as the geometric average of the subgraph edge weights (see details in reference [23]).

#### Assortativity

In network science theory, assortativity or assortative mixing is a network-based measure used to quantify the preference for a network’s nodes to link to other nodes that have similar degrees. In this paper, if we rank the institutions by their number of collaborations with others (i.e., the degree), the assortativity is the tendency for an institution to collaborate with other institutions with similar rank. There are several ways to capture such a correlation. A convenient approach is to use the Pearson correlation coefficient between the degrees found at the two ends of the same link. In our collaboration network, the Pearson correlation coefficient of weighted degree (the degree of institution *i* is defined as *s*_*i*_ = ∑_*j*_*w*_*ij*_) between pairs of linked nodes measures the similarity of connections in the graph with respect to the node degree, the value lies between − 1 and 1. Negative values mean that the links in the network tend to form between nodes with very different degrees, while positive values mean that links tend to form between nodes with similar degrees [24, 25].

#### Fraction of top institutions with 90% or greater collaboration weights

We rank the institutions according to their weighted degree. The degree of institution *i* is defined as *s*_*i*_ = ∑_*j*_*w*_*ij*_. We then calculate the minimal fraction of top institutions that account for 90% of the whole nodes’ degrees in the network. This measure captures the “dominant role” of top institutions, only a small fraction of top institutions account for more than 90% of the total collaborations in drug development; which is a common phenomenon observed in social science, economics, and network science.

## Supplementary information

**Additional file 1: Figure S1.** Citation growth after excluding self citations. **Figure S2.** PDE5 (phosphodiesterase type 5) inhibitors network analysis. **Figure S3.** HMG-CoA (hydroxymethylglutaryl (HMG)-CoA reductase) reductase inhibitors network analysis. **Figure S4.** TNF (tumor necrosis factor) (−alpha) inhibitors network analysis.

## Data Availability

All data and codes used in this study are free and available at https://github.com/yifangma/Collaboration_DrugDiscovery.

## Abbreviations

FDA: Food and Drug Administration
HMG-CoA: Hydroxymethylglutaryl (HMG)-CoA reductase
LDL-c: Low-density lipoprotein cholesterol
MAG: Microsoft Academic Graph
MeSH: Medical Subject Headings
PCSK9: Proprotein convertase subtilisin/kexin type 9
PDE5: Phosphodiesterase type 5
TNF: Tumor necrosis factor
