# Supplementary material for: Importance of scientific collaboration in contemporary drug discovery and development: a detailed network analysis
Source: BMC Biol. 2020 Oct 13;18:138. doi: 10.1186/s12915-020-00868-3 (PMC7556984; doi:10.1186/s12915-020-00868-3)
Supplement: Supplementary file 1 — Additional file 1: Figure S1. Citation growth after excluding self citations. Figure S2. PDE5 (phosphodiesterase type 5) inhibitors network analysis. Figure S3. HMG-CoA (hydroxymethylglutaryl (HMG)-CoA reductase) reductase inhibitors network analysis. Figure S4. TNF (tumor necrosis factor) (−alpha) inhibitors network analysis. [file 12915_2020_868_MOESM1_ESM.pdf]

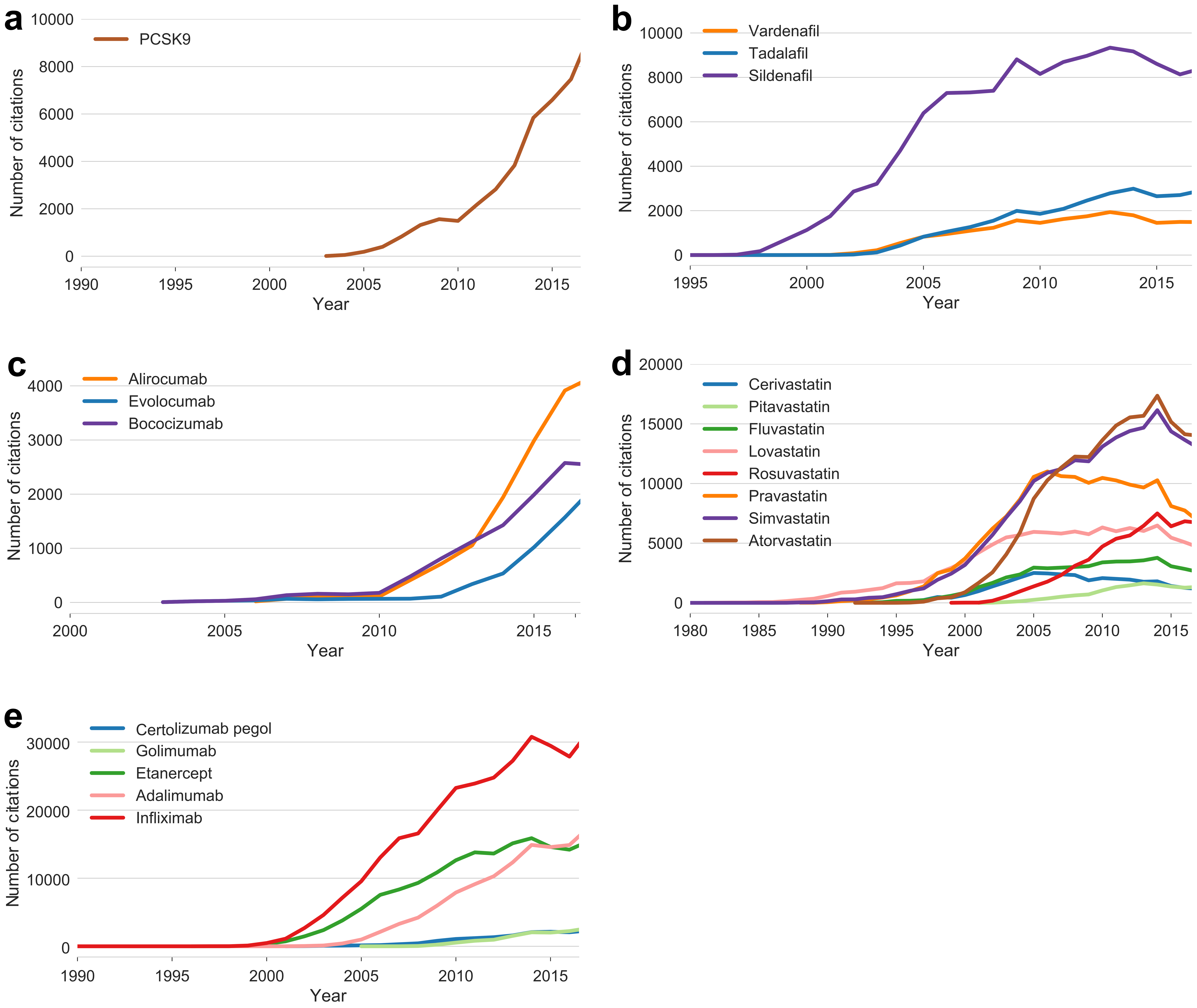


**Supplementary Fig. S1. Citation growth after excluding self citations. (a)** PCSK9 gene discovery and development; (**b**) 3 PCSK9 inhibitors (Alirocumab, Evolocumab and Bococizumab; (**c**) 3 PDE5 inhibitors (Vardenafil, Tadalafil, and Sildenafil); (**d**) 8 HMG-CoA reductase inhibitors (Cerivastatin, Pitavastatin, Fluvastatin, Lovastatin, Rosuvastatin, Pravastatin, Simvastatin and Atorvastatin); and (**e**) 5 TNF inhibitors (Certolizumab pegol, Golimumab, Etanercept, Adalimumab and Infliximab). In total, 170,099,684 publications dating from 1900 to 2017 were analyzed (see Supplementary Methods).

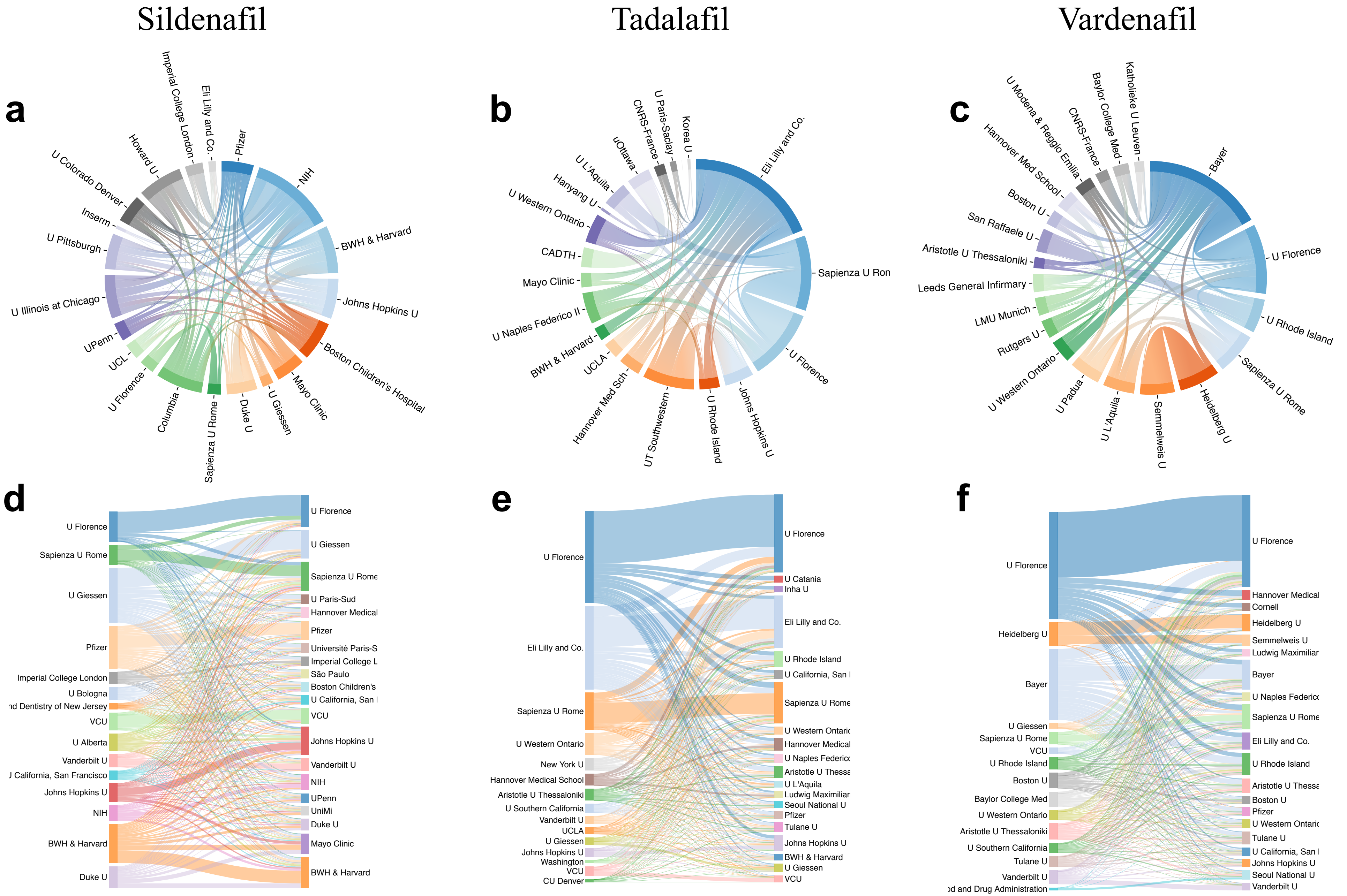


**Supplementary Fig. S2. PDE5 inhibitors network analysis. a-c,** Collaboration network for the top 20 institutions. Stripe width between institutions corresponds to the collaboration strength. **d-f,** The citation flow for the top institutions. Stripe width from institutions on the left to institutions on the right corresponds to the number of cases in which papers from institutions on the left were cited by papers from institutions on the right.

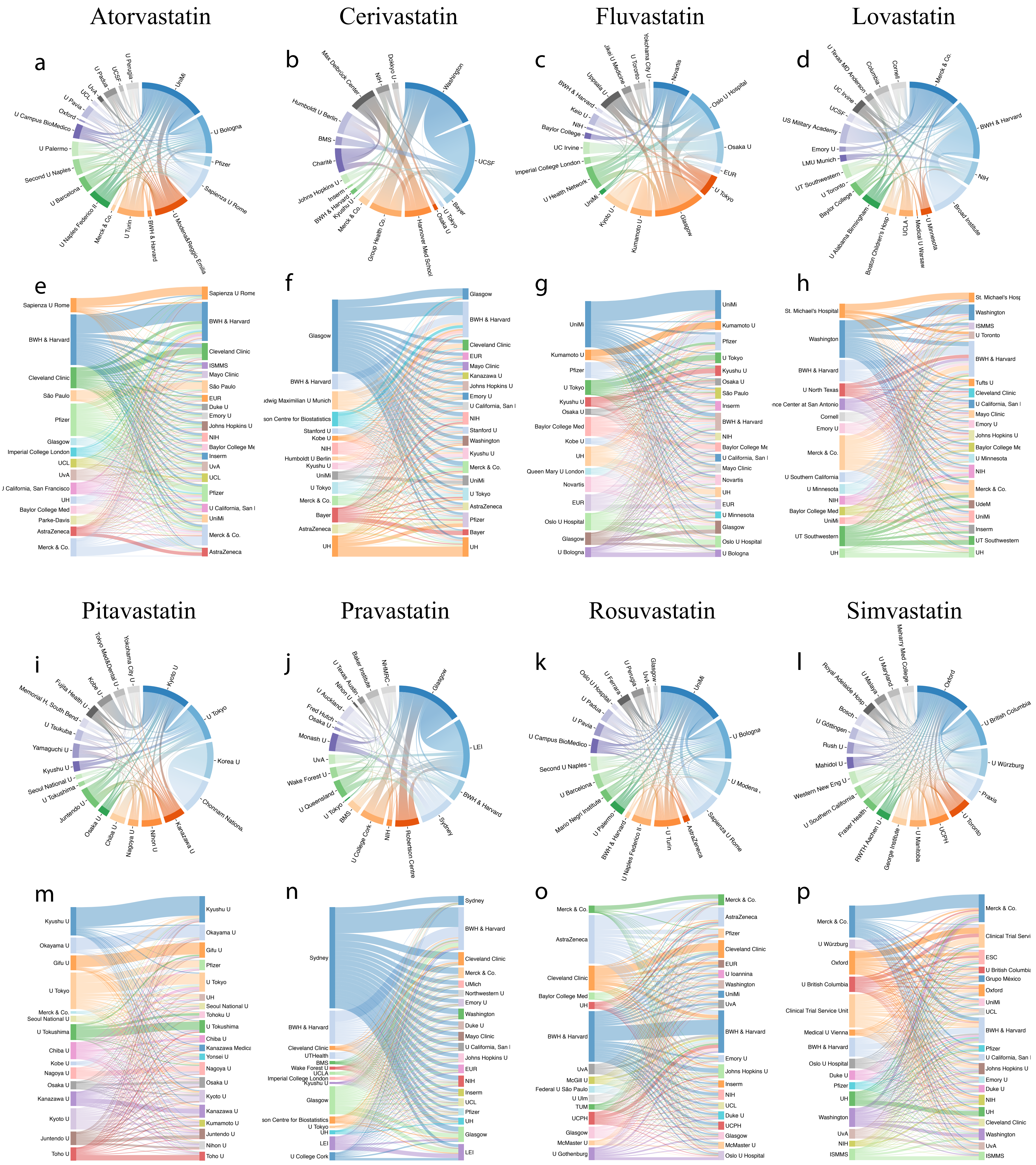


**Supplementary Fig. S3. HMG-CoA reductase inhibitors network analysis. a-d and i-l,** Collaboration network for the top 20 institutions. Stripe width between institutions corresponds to the collaboration strength. **e-h and m-p,** The citation flow for the top institutions. Stripe width from institutions on the left to institutions on the right corresponds to the number of cases in which papers from institutions on the left were cited by papers from institutions on the right.

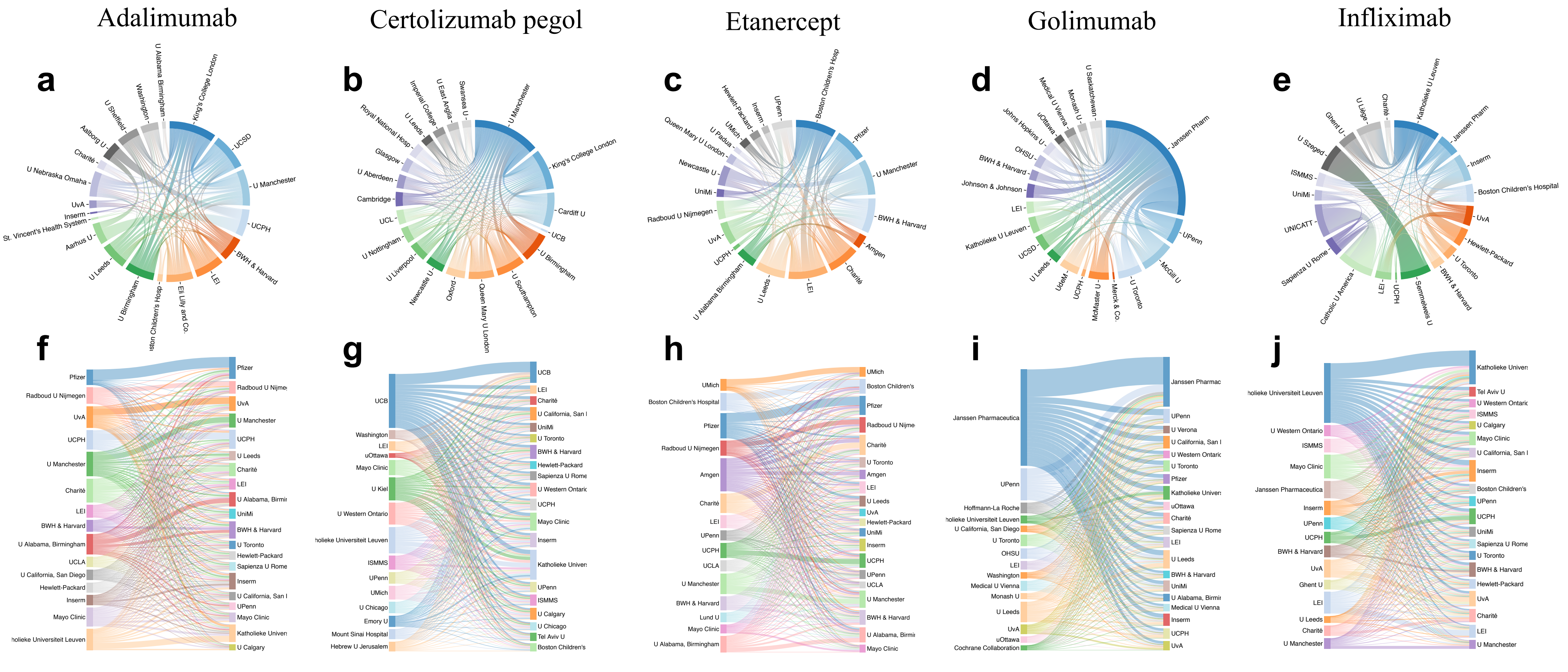


**Supplementary Fig. S4. TNF inhibitors network analysis. a-e,** Collaboration network for the top 20 institutions. Stripe width between institutions corresponds to the collaboration strength. **f-j,** The citation flow for the top institutions. Stripe width from institutions on the left to institutions on the right corresponds to the number of cases in which papers from institutions on the left were cited by papers from institutions on the right.
